# Effects of Multivitamin Supplementation on Metabolic Parameters in High- and Low-Fat Diet-Fed C57BL/6J Mice: Potential Links to Adipose Tissue Browning and Gut Microbiome

**DOI:** 10.3390/nu17061045

**Published:** 2025-03-17

**Authors:** Mehrnaz Abbasi, Braeden Heath, Lauren McGinness

**Affiliations:** 1 Department of Nutritional Sciences, College of Human Sciences, Auburn University, Auburn, AL 36849, USA; 2 Department of Biomedical Sciences, College of Sciences and Mathematics, Auburn University, Auburn, AL 36849, USA

**Keywords:** multivitamin supplementation, obesity, metabolic health, gut microbiota, adipose tissue browning, high-fat diet, low-fat diet, C57BL/6J mouse

## Abstract

Background/Objectives: The relationship between diet, micronutrient supplementation, and metabolic regulation emphasizes the potential of nutritional strategies to address obesity and related disorders. Certain vitamins have the potential to enhance thermogenesis and metabolic health. However, the impact of multivitamin supplementation on white adipose tissue (WAT) browning, the gut microbiome (GM), and metabolic function is not well understood. This study investigated the effects of multivitamin supplementation on obesity-related metabolic dysfunction in mice fed a high-fat diet (HFD) or a low-fat diet (LFD). Methods: Male C57BL/6J mice were assigned to group 1: control chow diet (CHD); 2: control HFD; 3: multivitamin-supplemented HFD (Mv-HFD); 4: control LFD; or 5: multivitamin-supplemented LFD (Mv-LFD). Diets, either supplemented with multivitamins A, D, B_1_, B_5_, and C or non-supplemented, were administered for 12 weeks. Metabolic parameters, adipose tissue browning, and the GM composition were analyzed. Results: The Mv-HFD significantly reduced weight gain, adipose tissue mass, blood glucose levels, and insulin resistance induced by an HFD. Additionally, it increased energy expenditure and thermogenic gene expression in WAT. Both the Mv-HFD and Mv-LFD improved the GM composition by increasing beneficial bacteria. Conclusions: Multivitamin supplementation improved metabolic health by potentially promoting WAT browning, enhancing energy expenditure, and modulating the GM composition. These findings suggest that multivitamins could offer a promising strategy for combating obesity and associated metabolic dysfunction.

## 1. Introduction

The complex relationship between dietary composition, nutritional interventions, and metabolic regulation highlights the important role of targeted nutritional strategies in understanding, addressing, and potentially preventing metabolic dysfunction and related chronic diseases, ultimately promoting better health and well-being [1,2,3,4]. It has been previously shown that dietary fat content can significantly affect various metabolic parameters [5,6,7]. Comparisons between high- and low-fat diets revealed notable differences in their effects on body weight, lipid and glucose metabolisms [7,8,9,10,11], and gut microbiome (GM) composition [11,12,13] in humans and mice. A high-fat diet (HFD) often leads to weight gain, fat accumulation, altered lipid profiles, impaired glucose tolerance, and changes in GM composition. In contrast, a low-fat diet (LFD) is associated with better weight management, improved insulin sensitivity, and a healthier GM profile [11,12,13]. However, the overall dietary context and other components can modulate these effects. Studies have demonstrated that the levels of micronutrients, such as vitamins, can interact with dietary fat to produce varied metabolic outcomes [14,15,16,17]. Therefore, a multi-nutrient approach is essential to fully elucidate the complex interplay between dietary components and their combined impact on metabolism.

Vitamins play a crucial role in managing obesity and related metabolic conditions. Research suggests that certain vitamins may help reduce body weight and offer anti-adipogenic, anti-inflammatory, antioxidant, and thermogenic benefits [16,18,19,20,21]. These effects are important for developing strategies to prevent and treat obesity and its complications, such as diabetes and cardiovascular diseases. However, the findings on the effects of vitamins have been inconsistent across studies [16]. Interestingly, while individual vitamins have been studied, there is a gap in the research regarding the effects of multivitamin supplements on metabolic health and adipose tissue metabolism. Understanding these effects and the underlying mechanisms could provide valuable insights for improving dietary interventions in obesity management.

Adipose tissue plays a crucial role in energy balance and metabolic health. The two main types are white adipose tissue (WAT) for energy storage and brown adipose tissue (BAT) for energy expenditure through thermogenesis. The “browning” of WAT, which takes on BAT-like characteristics, has emerged as a potential obesity treatment strategy due to its ability to increase energy expenditure [22]. Research focuses on identifying molecules that can activate BAT or induce WAT browning, as these processes enhance fatty acid oxidation and improve glucose homeostasis. Recent studies suggest that certain vitamins may aid in obesity management by promoting BAT activation and WAT browning [23,24]. Retinoic acid (RA), the carboxylic form of vitamin A, and 1α, 25-dihydroxy vitamin D_3_, the active hormonal form of vitamin D, have been shown to increase the expression of thermogenic genes in WAT and promote the preferential utilization of lipids as an energy source. Similarly, vitamins B_1_ (thiamine) and B_5_ (pantothenic acid) have demonstrated the ability to enhance thermogenesis in WAT. While vitamin C (ascorbate) has been shown to induce thermogenesis in interscapular BAT, its potential for inducing the browning of WAT has not been examined [20,21,25,26,27,28,29,30,31,32,33,34,35,36,37,38]. While some studies indicate a potential link between multivitamin intake and reduced obesity risk [18,39,40], the effects of multivitamin supplementation on WAT browning and its anti-obesity mechanisms remain unclear, warranting further investigation.

On the other hand, dietary components such as vitamins can modulate the composition and function of the GM, potentially impacting body weight regulation and adipose tissue function [41,42]. This dynamic interaction highlights the essential role of nutrition in promoting gut health and, consequently, overall metabolic well-being.

This study investigated the effects of dietary multivitamin supplementation (A, D, B_1_, B_5_, and C) with thermogenic potential on obesity-related metabolic parameters, WAT browning, and GM composition in mice. The experiment compared mice fed an HFD and LFD supplemented with multivitamins to their respective control groups without supplementation. The aim was to assess whether this dietary approach could improve metabolic health and understand the mechanisms linking these benefits to WAT browning and GM changes.

## 2. Materials and Methods

### 2.1. Animals and Experimental Designs

Male C57BL/6J mice from Jackson Laboratory at 4 weeks of age were housed at 22–24 °C, 45% relative humidity, a daily 12 h light/dark cycle, and with free access to water and food. After 1 week of acclimation, the mice were weighed and randomly assigned to one of the following treatments for 12 weeks: 1. Control chow diet (CHD, 18% fat, no supplementation), 2. Control high-fat diet (HFD, 45% fat, no supplementation), 3. Multivitamin-supplemented HFD (Mv-HFD, 45% fat, supplemented with vitamins A, D, B_1_, B_5_, and C), 4. Control low-fat diet (LFD, 10% fat, no supplementation), or 5. Multivitamin-supplemented LFD (Mv-LFD, 10% fat, supplemented with vitamins A, D, B_1_, B_5_, and C). The diets were developed and produced by Research Diets, Inc., New Brunswick, NJ, USA. The chow diet used was Teklad 2918X from Inotiv, Inc., Lafayette, IN, USA. Details about the compositions of the custom diets can be found in Supplementary Table S1. Food intake and body weights were recorded weekly. The glucose tolerance test (GTT), indirect calorimetry, and cold tolerance test were performed in the two final weeks of treatment. The mice were fasted overnight and euthanized humanely at the end of the treatments. Blood was collected from the abdominal vein, and serum was obtained via centrifugation at 2000× *g* at 4 °C for 30 min. The major organs and tissues of each mouse were collected for analysis. The animal work was approved by the committee on animal care (Auburn University, protocol 2023–5335). A sample size of n = 5 per group was used in this study.

### 2.2. GTT and Homeostatic Model Assessment for Insulin Resistance (HOMA-IR)

For the GTT, the mice were fasted for 6 h before receiving an intraperitoneal injection of glucose (2.5 g/kg of body weight). Blood was sampled from the tail vein immediately before the injection (0 min) and at 15, 30, 60, 90, and 120 min afterward. A One Touch^®^ glucometer determined the blood glucose levels at each time point. The glucose response was analyzed by graphing the blood glucose concentration versus time. To quantify the overall glycemic response, the area under the curve (AUC) was calculated using the following equation: AUC = ((C1 + C2)/2) × (T2 − T1). In this formula, C denotes the glucose concentration (mg/dL), and T represents the time (minutes). This calculation was performed for each pair of adjacent time points and summed to obtain the total AUC for the GTT. HOMA-IR was calculated using the following formula: HOMA-IR = [fasting glucose (mmol/L) × fasting insulin (μU/mL)]/22.5.

### 2.3. Indirect Calorimetry

Metabolic parameters were evaluated using the Promethion Metabolic and Behavioral Phenotyping System (Sable Systems International, Las Vegas, NV, USA). The system measured oxygen consumption (VO_2_ [mL/h/kg]), carbon dioxide production (VCO_2_ [mL/h/kg]), the respiratory exchange ratio (RER, calculated as VCO_2_/VO_2_), and energy expenditure (EE [kcal/h/kg]) through indirect calorimetry. Before data collection, the mice were acclimated to the cages for 24 h. The experiment was conducted under a 12 h light/dark cycle, with animals having unrestricted access to food and water. Measurements were recorded over a subsequent 48 h period.

### 2.4. Cold Tolerance Test

Before the experiment, each mouse’s initial rectal temperature (T_Core_) was recorded. Then, a modified version of the cold sensitivity assessment protocol developed by Brenner D.S. et al. [43] was implemented. The mice were individually housed in bedding-free cages with free access to food and water. Cages were placed on glass plates cooled to 4 °C using aluminum boxes filled with dry ice and monitored with a thermocouple. Cages were also surrounded by similar aluminum boxes filled with dry ice to maintain the temperature at 4 °C for 4 h. Throughout this period, we measured the rectal temperatures at hourly intervals. A thermometer was inserted ~2–2.5 mm into the rectum for each measurement. Between measurements, the rectal probe was disinfected with alcohol wipes and lubricated with glycerol.

### 2.5. Infrared (IR) Thermography

The surface (skin) temperature (T_Skin_) was measured using IR thermography, a non-invasive technique that converts IR radiation from an object’s surface into a color-coded image. For the measurements, the mice were placed individually into clean, bedding-free cages or on non-reflective surfaces at room temperature or during a cold tolerance test. A HIKMICRO B20 thermal camera (Hangzhou Microimage Intelligent Technology Co., Ltd., Hangzhou, China) was positioned 50–100 cm above the mouse to capture thermal images focusing on the body surface.

### 2.6. Histological Analysis

Liver and inguinal WAT (IWAT) samples were collected during necropsy and fixed in 10% neutral buffered formalin. They underwent a 12 h processing cycle using a Leica TP1040 (Leica Microsystems, Inc., Deerfield, IL, USA). This process involved immersing the samples in 70% ethanol for 1 h, followed by 1 h in 80% ethanol, three stations of 95% ethanol for 1 h each, and three stations of 100% ethanol for 1 h each. The samples were then treated in two stations of xylene substitute for 1 h each, followed by two stations of liquefied paraffin at 60 °C for 1 h each. After processing, the samples were embedded in StatLab Parapro 360 paraffin using a Sakura Tissue Tek IV embedding station (Sakura Finetek, Inc., Torrance, CA, USA) and sectioned at 4 μm with a Leica 2125 RM microtome (Leica Microsystems Inc., Deerfield, IL, USA). The sections were air-dried overnight, baked at 60 °C for 10 min, and manually stained with Hematoxylin and Eosin (H&E) solutions. Finally, the slides were cover slipped with CytoSeal XYL mounting medium (Epredia, Inc., Kalamazoo, MI, USA), and histological images were captured using an Olympus VS200 Slide Scanner (Olympus Corporation, Center Valley, PA, USA).

### 2.7. Adipocyte Size

Adipocyte size quantification was performed by capturing images of H&E-stained histological sections of IWAT from mice using the Olympus VS200 Slide Scanner (Olympus Corporation, Center Valley, PA, USA). Image J software (version 1.51) was used for the fat cell area measurements.

### 2.8. Serum Biochemical Analysis of Insulin and Lipid Profiles

Serum insulin concentrations, total cholesterol, triglycerides, LDL, and HDL were measured using the RX Daytona+ analyzer with Randox product inserts (Randox Laboratories, Ltd., Kearneysville, WV, USA). Serum insulin was measured using an in-house developed rodent Insulin Radioimmunoassay, modified from the rat insulin RIA kit (MilliporeSigma, Burlington, MA, USA) and Linco Research, Inc. (Charles, MO, USA) assay procedure. All assays were conducted at the Michigan Diabetes Research Center (MDRC) Chemistry Laboratory (Ann Arbor, MI, USA).

### 2.9. Quantitative Real-Time PCR Analysis

RNA was isolated from IWAT and liver samples using TRIzol^®^ reagent from Invitrogen Inc. (Waltham, MA, USA). The extracted RNA was then quantified to synthesize first-strand cDNA using the Maxima First Strand cDNA Synthesis Kit (Thermo Fisher Scientific, Inc., Waltham, MA, USA). Gene expression was analyzed via quantitative PCR using the PowerUp SYBR™ Green Master Mix (Thermo Fisher Scientific, Inc., Waltham, MA, USA) on a QuantStudio™ 3 Real-Time PCR System (Applied Biosystems, Inc., Foster City, CA, USA). Primers were purchased from Integrated DNA Technologies, Inc. (Coralville, IA, USA). The relative expression of target genes was calculated using the 2^−ΔΔCt^ method, with 36B4 or β-actin serving as internal controls for normalization. A list of primer sequences can be found in Supplementary Table S2.

### 2.10. Immunohistochemistry

IWAT samples were collected during necropsy and fixed in 10% neutral buffered formalin. A 22 h processing cycle was conducted using a Leica TP1040 (Leica Microsystems, Inc., Deerfield, IL, USA) processing unit, involving immersion in 70% ethanol for 2 h, followed by 80%, 95% (three times), and 100% ethanol (twice), with one additional 100% ethanol treatment for 1.5 h. The samples were then treated in two stations of a xylene substitute for 1.5 h each, followed by two liquefied paraffin stations at 60 °C for 2 h each. The samples were embedded in StatLab Parapro 360 paraffin using a Sakura Tissue Tek IV embedding station (Sakura Finetek, Inc., Torrance, CA, USA) and sectioned at 4 μm with a Leica 2125 RM microtome (Leica Microsystems Inc., Deerfield, IL, USA). The sections were placed on Matsunami MAS-GP adhesion slides (Matsunami Glass USA, Inc., Bellingham, WA, USA) and air-dried overnight. Before staining, the slides were baked at 60 °C for 15 min and stained using a Leica Bond RXm autostainer (Leica Microsystems, Inc., Deerfield, IL, USA) with the Leica Bond Polymer Refine Detection kit (Leica Biosystems, Buffalo Grove, IL, USA). After staining, the slides were cleared in 95% ethanol for 2 min, 100% ethanol for 2 min, and xylene for 2 min before cover slipping. Anti-*Ucp1* antibody were purchased from MilliporeSigma, Inc. (Burlington, MA, USA). Vectastain ABC kits were purchased from Vector Laboratories (Burlingame, CA, USA). The slides were captured and visualized using an Olympus VS200 Slide Scanner (Olympus Corporation, Center Valley, PA, USA).

#### 2.11. 16S rRNA Analysis and Bioinformatics

Following a previously established protocol, we conducted 16S rRNA analysis and bioinformatics on fecal samples at the University of Alabama at Birmingham Microbiome Center (Birmingham, AL, USA) [44]. Following the manufacturer’s instructions, microbial genomic DNA was extracted using the Zymo Research Fecal DNA Isolation Kit (Zymo Research Corporation, Irvine, CA, USA). The variable region 4 (V4) of the 16S rDNA gene was amplified via PCR with sample-specific barcoded primers to create an amplicon library for each sample. The resulting PCR product, approximately 255 base pairs in length from the V4 region, was sequenced using Illumina MiSeq technology (Illumina, San Diego, CA, USA), generating single-end reads of 251 base pairs. The sequencing results were analyzed using USEARCH v6.1 software, and operational taxonomic units (OTUs) were obtained by clustering at a 97.0% similarity level. The raw data files were converted to FASTQ format after demultiplexing with MiSeq Reporter. Quality control of the sequence reads was performed using DADA2, which filtered out low-quality data based on specific parameters in the fastqPairedFilter function. DADA2 was also utilized for denoising and clustering reads into Amplicon Sequence Variants. Taxonomic assignments were made using Mothur with the SILVA 16S rDNA database (SILVA_132_QIIME_release). These processes were integrated into an updated version of the automated analysis pipeline known as QWRAP (Quantitative Insights Into Microbial Ecology (QIIME) wrapper). QIIME was employed to calculate various diversity metrics, including α-diversity (Shannon, Simpson, PD whole tree, and observed species) and β-diversity (Bray–Curtis and weighted/unweighted UniFrac). Principal coordinates analysis (PCoA) was performed using UniFrac distances to conduct community structure UniFrac distance-based Analysis of Similarity (ANOSIM) to assess clustering patterns.

### 2.12. Statistical Analysis

Statistical analyses were performed using IBM SPSS Statistics for Windows, Version 25.0 (IBM Corp., Armonk, NY, USA), R version 1.3.1 (R Foundation for Statistical Computing, Vienna, Austria), and figures were created with GraphPad Prism version 10.0.0 (GraphPad Software, San Diego, CA, USA). The data are presented as the mean ± standard deviation. Student’s *t*-test was employed for pairwise group comparisons, while ANOVA with Tukey’s post hoc correction was utilized for multiple comparisons. A *p*-value of less than 0.05 was considered statistically significant.

## 3. Results

### 3.1. Food Intake Varied with Dietary Fat Content

**Figure 1:**
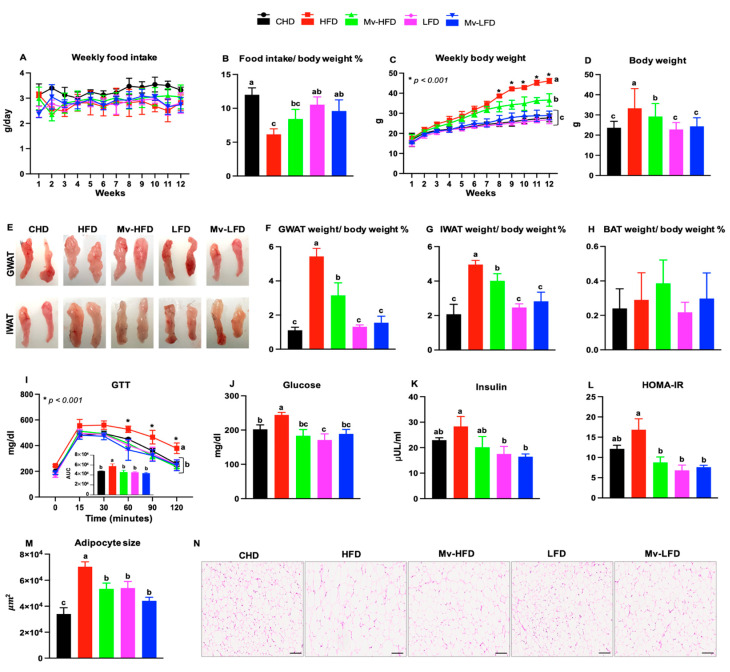
Mv-HFD decreased HFD-induced increases in mice’s body weight, GWAT and IWAT weights, blood glucose, HOMA-IR, and adipocyte size. (**A**) Weekly food intake, (**B**) % body weight normalized food intake, (**C**) weekly body weight, (**D**) body weight, (**E**) representative images of GWAT and IWAT, % body weight normalized (**F**) GWAT, (**G**) IWAT, and (**H**) BAT weights, (**I**) GTT and GTT-AUC, (**J**) blood glucose, (**K**) serum insulin, (**L**) HOMA-IR, (**M**), adipocyte size, and (**N**) representative H&E images of IWAT adipocytes (Scale bar: 200 µm). In subfigures (**C**,**I**), * *p *< 0.001: HFD group compared to CHD, Mv-HFD, LFD, and Mv-LFD. n = 5, data are presented as the mean ± SD. Bars or lines without a common superscript differ, *p *< 0.05 via one-way ANOVA followed by Tukey’s HSD post hoc test.

The weekly food intake was monitored to determine the effects of dietary fat content and vitamin supplementation during the intervention (Figure 1A). Average food intake was normalized to body weight. As shown in Figure 1B, HFD-treated mice had significantly lower food intake than the CHD (1.94-fold, *p* < 0.001), LFD (1.71-fold, *p* < 0.001), and Mv-LFD (1.55-fold, *p* = 0.004) treatment groups. The Mv-HFD-treated mice had significantly lower food intake than the CHD-treated mice (1.42-fold, *p* = 0.003). Food intake was similar between the HFD and Mv-HFD groups and among the Mv-HFD, LFD, and Mv-LFD groups.

### 3.2. Mv-HFD Decreased HFD-Induced Body Weight Gain

Body weight gain was monitored weekly for 12 weeks to assess the impacts of dietary fat and vitamin supplementation (Figure 1C). The Mv-HFD-treated mice gained significantly less body weight than the HFD-treated mice (1.32-fold, *p* < 0.001). While both the Mv-HFD- and HFD-treated mice gained more weight than the CHD, LFD, and Mv-LFD groups, the weight gain in the Mv-HFD-treated mice was significantly less (*p *< 0.001). Specifically, the Mv-HFD mice showed a smaller fold increase in weight compared to the HFD mice relative to the CHD (1.32 vs. 1.66), LFD (1.37 vs. 1.72), and Mv-LFD (1.26 vs. 1.59) groups. However, there was no significant difference in body weight among the CHD, LFD, and Mv-LFD groups (Figure 1C,D).

### 3.3. Mv-HFD Decreased HFD-Induced Increases in Gonadal WAT (GWAT) and IWAT Weights

Representative images of GWAT and IWAT are shown in Figure 1E. The Mv-HFD-treated mice exhibited significantly lower weights in both GWAT (1.32-fold, *p* < 0.05) and IWAT (1.72-fold, *p* < 0.001) (normalized to body weight) compared to the HFD-treated mice, with more pronounced differences than those observed between the HFD and other groups, as evidenced by the fold changes in GWAT (CHD: 1.93 vs. 2.39, LFD:1.62 vs. 2.01, and Mv-LFD: 1.41 vs. 1.75, respectively) and IWAT (CHD: 2.84 vs. 4.90, LFD: 2.40 vs. 4.14, and Mv-LFD: 2.02 vs. 3.50, respectively) (Figure 1F,G). However, the BAT weights were not significantly different among treatment groups (Figure 1H).

### 3.4. Mv-HFD Decreased HFD-Induced Increases in Blood Glucose and HOMA-IR

Given the reduced body weight and fat mass in the Mv-HFD mice, we examined whether this led to improved glucose tolerance and insulin sensitivity by performing GTTs and HOMA-IR tests. The HFD-treated mice had a significantly (*p* < 0.001) higher GTT-AUC than mice in the CHD (1.2-fold), Mv-HFD (1.26-fold), LFD (1.27-fold), and Mv-LFD (1.32-fold) treatment groups. The Mv-HFD treatment significantly (*p *< 0.001) reduced the GTT values at 30, 60, 90, and 120 min time points compared to the HFD treatment, with reductions of 1.13-, 1.26-, 1.45-, and 1.61-fold, respectively. However, no significant difference existed among the mice in the CHD, Mv-HFD, LFD, and Mv-LFD treatment groups (Figure 1I).

The Mv-HFD and Mv-LFD treatments resulted in significantly (*p *< 0.001) lower blood glucose levels compared to the HFD (1.20- and 1.33-fold, respectively). In comparison, the LFD-treated mice also showed significantly (*p *< 0.05) lower blood glucose levels than both the CHD (1.18-fold) and HFD (1.42-fold) groups (Figure 1J).

The LFD- and Mv-LFD-treated mice showed significantly (*p* < 0.05) lower serum insulin levels than mice in the HFD group, with 1.61-fold and 1.72-fold reductions, respectively. However, no significant differences were observed among the other treatment groups (Figure 1K).

HOMA-IR was significantly (*p* < 0.005) lower in mice treated with an Mv-HFD (1.92-fold), LFD (2.46-fold), and Mv-LFD (2.21-fold) compared to those in the HFD group. However, no significant differences were observed between the CHD and other treatment groups (Figure 1L).

### 3.5. Mv-HFD Decreased HFD-Induced Increases in Adipocyte Size in IWAT

Next, we investigated the effects of dietary fat content and vitamin supplementation on adipocyte size in IWAT. The adipocyte size was significantly (*p *< 0.005) smaller in mice treated with the CHD (2.06-fold), Mv-HFD (1.31-fold), LFD (1.30-fold), and Mv-LFD (1.59-fold) compared to those in the HFD group. Among these groups, the CHD-treated mice exhibited the smallest adipocyte size, which was significantly (*p *< 0.01) lower than the Mv-HFD (1.56-fold), LFD (1.58-fold), and Mv-LFD (1.29-fold) groups. (Figure 1M). Representative H&E images of IWAT adipocytes are shown in Figure 1N.

### 3.6. Mv-HFD Enhanced HFD-Induced Reductions in Rectal and Surface Temperatures

**Figure 2:**
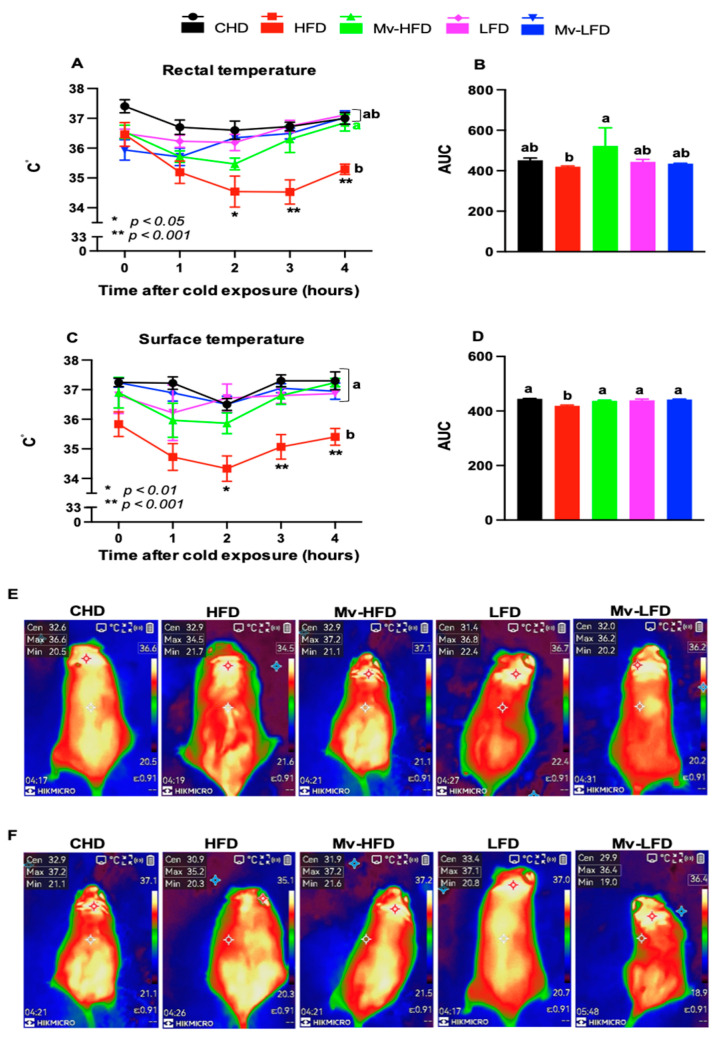
Mv-HFD enhanced HFD-induced reductions in rectal and surface temperatures in mice. (**A**) Rectal temperature (T_Core_), (**B**) T_Core_-AUC, (**C**) surface temperature (T_Skin_), (**D**) T_Skin_-AUC, (**E**) representative images of T_Skin_ at room temperature, and (**F**) representative images of T_Core_ at 4 °C. In subfigures (**A**,**C**), * *p* < 0.05, and ** *p *< 0.001: HFD group compared to CHD, Mv-HFD, LFD, and Mv-LFD. n = 5, data are presented as the mean ± SD. Bars or lines without a common superscript differ, *p *< 0.05 via one-way ANOVA followed by Tukey’s HSD post hoc test. SD. Bars or lines without a common superscript differ, *p *< 0.05 via one-way ANOVA followed by Tukey’s HSD post hoc test.

T_Core_ and T_Skin_ were measured as indicators of metabolic regulation. After 4 h of cold exposure, HFD-treated mice exhibited significantly lower T_Core_ (*p *< 0.05) and T_Skin_ (*p *< 0.01) compared to mice in other groups, with fold differences of ~1.05 for both parameters (Figure 2A,C). Furthermore, the T_Core_-AUC was significantly (*p *< 0.05) higher in mice treated with the Mv-HFD (1.24-fold), LFD (1.06-fold), and Mv-LFD (1.03-fold) compared to the HFD group (Figure 2B). The T_Skin_-AUC was significantly (*p *< 0.001) higher in the Mv-HFD- (1.04-fold), LFD- (1.05-fold), and Mv-LFD-treated (1.05-fold) mice compared to the HFD group (Figure 2D).

Representative IR-thermography images of T_Skin_ recorded at room and 4 °C temperatures are shown in Figure 2E,F. HFD-treated mice exhibited lower T_Skin_ than mice in other treatment groups at room temperature and after 4 h at 4 °C. At room temperature, the differences were 2.1 °C, 2.7 °C (*p *< 0.05), 2.3 °C, and 1.7 °C lower than the CHD, Mv-HFD, LFD, and Mv-LFD groups, respectively (Figure 2E). Similarly, at 4 °C, the HFD-treated mice had 2.0 °C, 2.0 °C (*p *< 0.05), 1.9 °C, and 1.2 °C lower T_Skin_ than the CHD, Mv-HFD, LFD, and Mv-LFD groups, respectively (Figure 2F).

### 3.7. Mv-HFD Decreased HFD-Induced Increases in Total Cholesterol and Liver Lipid Accumulation

**Figure 3:**
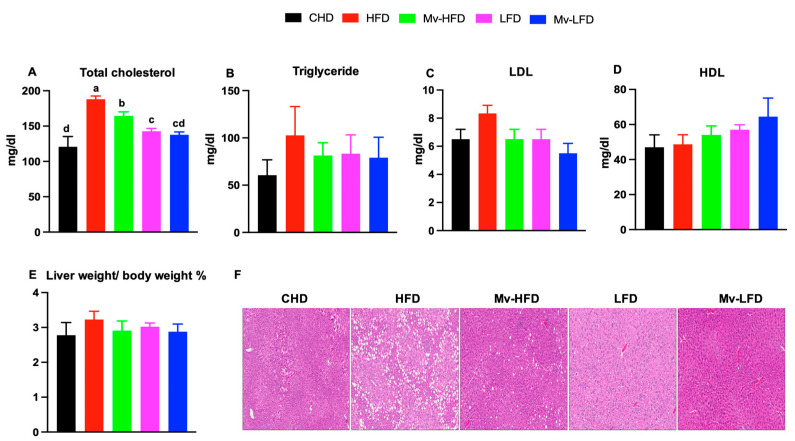
Mv-HFD decreased HFD-induced increases in total cholesterol and liver lipid accumulation in mice. (**A**) Total cholesterol, (**B**) triglyceride, (**C**) LDL, (**D**) HDL, (**E**) % body weight normalized liver weight, and (**F**) representative H&E images of the liver. n = 5, data are presented as the mean ± SD. Bars or lines without a common superscript differ, *p *< 0.05 via one-way ANOVA followed by Tukey’s HSD post hoc test.

We measured the lipid profile in mice to assess the effects of dietary fat content and vitamin supplementation on lipid metabolism. The HFD-treated mice exhibited significantly higher total cholesterol levels compared to the other groups: CHD (1.55-fold, *p *< 0.001), Mv-HFD (1.14-fold, *p *= 0.024), LFD (1.31-fold, *p *< 0.001), and Mv-LFD (1.36-fold, *p *< 0.001) (Figure 3A). Although the HFD-treated mice also showed higher triglyceride and LDL levels compared to other groups, these differences were not statistically significant (Figure 3B,C). Mice treated with an Mv-HFD, LFD, and Mv-LFD showed higher HDL levels than HFD-treated mice, but these differences were not statistically significant (Figure 3D).

We analyzed liver weights and performed H&E staining on liver sections to assess lipid accumulation further. The liver weight (normalized to body weight) was higher in the HFD-treated mice compared to the CHD (1.20-fold), Mv-HFD (1.11-fold), LFD (1.07-fold), and Mv-LFD (1.12-fold) groups. However, these differences were not statistically significant (Figure 3E). Representative H&E images of the liver, shown in Figure 3F, illustrate lipid accumulation. The HFD-treated mice exhibited a greater accumulation of lipid droplets within liver cells, while the Mv-HFD-treated mice showed significantly less accumulation.

### 3.8. Mv-HFD Increased VO_2_, VCO_2_, and EE and Reduced RER

**Figure 4:**
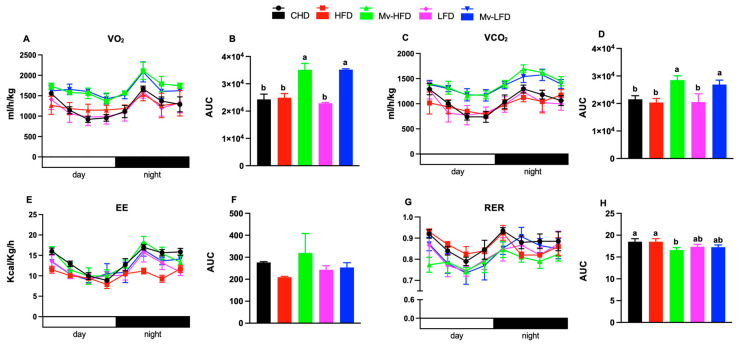
Mv-HFD increased VO_2_, VCO_2_, and EE and reduced mice’s RER. (**A**,**B**) O_2_ consumption (VO_2_) and the area under the curve (AUC), (**C**,**D**) CO_2_ production (VCO_2_) and AUC, (**E**,**F**) energy expenditure (EE) and AUC, (**G**,**H**) respiratory exchange ratio (RER) and AUC. n = 4–5, data are presented as the mean ± SD. Bars or lines without a common superscript differ, *p *< 0.05 by one-way ANOVA followed via Tukey’s HSD post hoc test.

We performed a metabolic cage to explore the effect of dietary fat content and vitamin supplementation on EE and RER in mice. The VO_2_-AUC was significantly increased (*p *< 0.01) in the Mv-HFD and Mv-LFD-treated mice compared to those in the CHD (1.44- and 1.45-fold), HFD (1.40- and 1.41-fold), and LFD (1.53- and 1.54-fold) groups (Figure 4A,B). The VCO_2_-AUC was significantly increased (*p *< 0.001) in the Mv-HFD- and Mv-LFD-treated mice compared to those in the CHD (1.32- and 1.24-fold), HFD (1.31- and 1.23-fold), and LFD (1.38- and 1.31-fold) groups (Figure 4C,D).

The EE-AUC was higher in the Mv-HFD-treated mice compared to those in the HFD (1.52-fold), LFD (1.35-fold), and Mv-LFD (1.26-fold) groups; however, the differences were not statistically significant (Figure 4E,F). The RER-AUC was significantly decreased (*p *< 0.02) in the Mv-HFD-treated mice, approaching an RER of 0.7 compared to mice in the CHD and HFD groups (1.11-fold for both), indicating a greater reliance on lipids for oxidative metabolism (Figure 4G,H).

### 3.9. Mv-HFD, LFD, and Mv-LFD Reduced Hepatic mRNA Expression of Srebp1c, Fas, Glut2, and Tnfα

**Figure 5:**
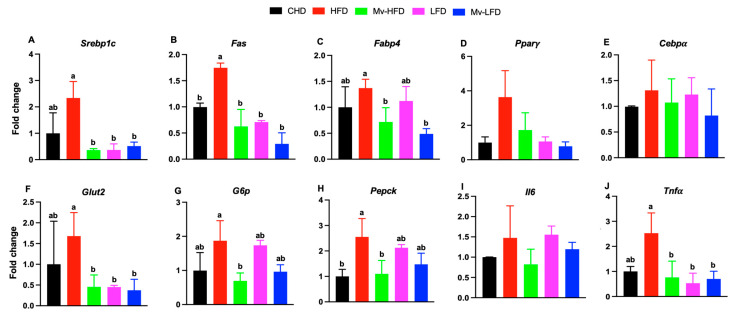
Mv-HFD, LFD, and Mv-LFD reduced hepatic mRNA expression of *Srebp1c*, *Fas*, *Glut2*, and *Tnfα* compared to HFD in mice. (**A**) *Srebp1c*, (**B**) *Fas*, (**C**) *Fabp4*, (**D**) *Pparγ*, (**E**) *Cebpα*, (**F**) *Glut2*, (**G**) *G6p*, (**H**) *Pepck*, (**I**) Il6, and (**J**) *Tnfα*. n = 5, data are presented as the mean ± SD. Bars without a common superscript differ, *p* < 0.05, via one-way ANOVA followed by Tukey’s HSD post hoc test.

Considering the differences in lipid profiles and hepatic lipid accumulation, we examined how dietary fat content and vitamin supplementation impact lipid and glucose metabolisms and the inflammatory response by analyzing the hepatic expression of key genes involved. Mice treated with an HFD showed significantly (*p* < 0.01) higher gene expression of *Srebp1c* compared to those in the Mv-HFD (6.47-fold), LFD (6.34-fold), and Mv-LFD (4.54-fold) groups (Figure 5A). Mice treated with an HFD exhibited significantly higher gene expression of *Fas* compared to those in the CHD (1.75-fold, *p *= 0.021), Mv-HFD (2.78-fold, *p *< 0.001), LFD (2.46-fold, *p *= 0.003), and Mv-LFD (5.96-fold, *p* < 0.001) groups (Figure 5B). Mice treated with an HFD showed significantly (*p *< 0.05) higher gene expression of *Fabp4* compared to those in the Mv-HFD (1.91-fold) and Mv-LFD (2.82-fold) groups (Figure 5C). Mice treated with an HFD exhibited higher gene expression of *Pparγ* compared to those in the CHD (3.65-fold), Mv-HFD (2.11-fold), LFD (3.41-fold), and Mv-LFD (4.64-fold) groups. However, these differences were not statistically significant (Figure 5D). Similarly, the HFD-treated mice showed higher gene expression of *Cebpα* compared to those in the CHD (1.32-fold), Mv-HFD (1.22-fold), LFD (1.06-fold), and Mv-LFD (1.60-fold) groups. However, these differences were not statistically significant (Figure 5E).

The HFD-treated mice exhibited significantly (*p* < 0.05) higher gene expression of *Glut2* compared to those in the Mv-HFD (3.68-fold), LFD (3.72-fold), and Mv-LFD (4.49-fold) groups (Figure 5F). *G6p* expression was higher in the HFD-treated mice than in the Mv-HFD group (2.69-fold, *p *= 0.03) (Figure 5G). Similarly, *Pepck* expression was significantly (*p* < 0.05) elevated in the HFD-treated mice compared to both the CHD (2.55-fold) and Mv-HFD (2.32-fold) groups (Figure 5H).

*Il6* expression was elevated in the HFD-treated mice compared to the CHD (1.47-fold), Mv-HFD (1.79-fold), and Mv-LFD (1.23-fold) groups, although these differences were not statistically significant (Figure 5I). In contrast, *Tnfα* expression was significantly (*p *< 0.03) higher in the HFD-treated mice compared to the Mv-HFD (3.32-fold), LFD (4.75-fold), and Mv-LFD (3.61-fold) groups (Figure 5J).

### 3.10. Mv-HFD and Mv-LFD Reduced IWAT mRNA Expression of Fabp4 and Tnfα

**Figure 6:**
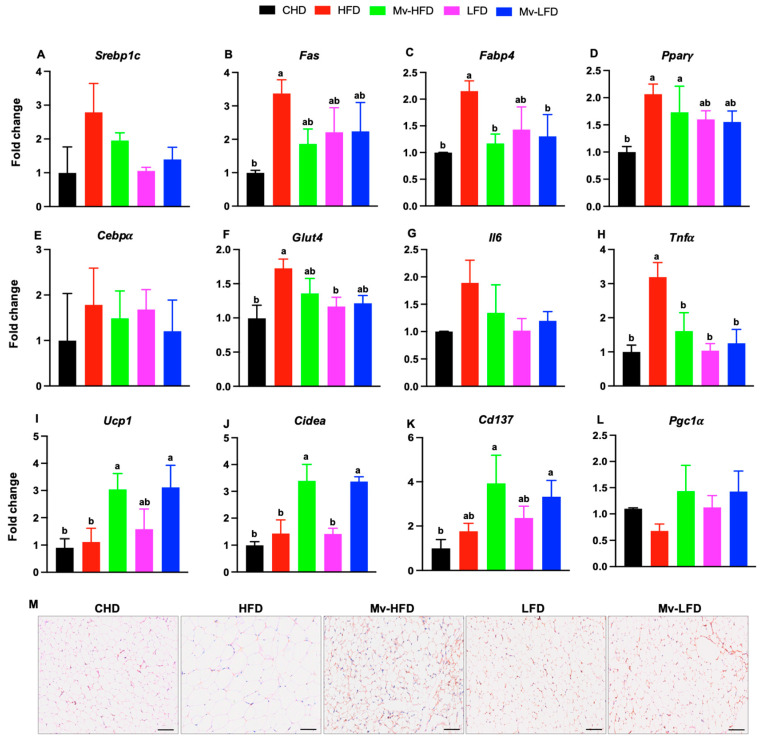
Mv-HFD and Mv-LFD reduced IWAT mRNA expression of *Fabp4* and *Tnfα* and increased *Ucp1*, *Cidea*, and *Cd137* compared to HFD in mice. (**A**) *Srebp1c*, (**B**) *Fas*, (**C**) *Fabp4*, (**D**) *Pparγ*, (**E**) *Cebpα*, (**F**) *Glut4*, (**G**) *Il6*, (**H**) *Tnfα*, (**I**) *Ucp1*, (**J**) *Cidea,* (**K**) *Cd137*, (**L**) *Pgc1α*, and (**M**) Representative immunohistochemistry staining (*Ucp1*) images of IWAT (Scale bar: 200 µm). n = 5, data are presented as the mean ± SD. Bars without a common superscript differ, *p* < 0.05, via one-way ANOVA followed by Tukey’s HSD post hoc test.

We investigated how dietary fat content and vitamin supplementation influence gene expression related to IWAT lipid and glucose metabolisms and inflammation. *Srebp1c* expression was elevated in the HFD-treated mice compared to the CHD (2.80-fold), Mv-HFD (1.43-fold), LFD (2.64-fold), and Mv-LFD (2.00-fold) groups, although these differences were not statistically significant (Figure 6A). Similarly, *Fas* expression was higher in the HFD-treated mice compared to the CHD (3.38-fold, *p *= 0.034), Mv-HFD (1.81-fold), LFD (1.52-fold), and Mv-LFD (1.50-fold) groups. However, the other comparisons were not statistically significant except for the significant difference with the CHD group (Figure 6B).

Mice treated with an HFD showed significantly (*p *< 0.05) higher gene expression of *Fabp4* compared to those in the CHD (2.15-fold), Mv-HFD (1.83-fold), and Mv-LFD (1.64-fold) groups (Figure 6C). Similarly, *Pparγ* expression was elevated in the HFD- and Mv-HFD-treated mice compared to the CHD group (3.65-fold, *p *= 0.001 and 1.73-fold, *p *= 0.032, respectively). However, the differences in *Pparγ* expression were not statistically significant among other groups (Figure 6D). Mice treated with an HFD exhibited higher gene expression of *Cebpα* compared to those in the CHD (1.79-fold), Mv-HFD (1.20-fold), LFD (1.06-fold), and Mv-LFD (1.48-fold) groups. However, these differences were not statistically significant (Figure 6E).

Mice treated with an HFD exhibited significantly (*p *< 0.05) higher gene expression of *Glut4* compared to those in the CHD (1.73-fold) and LFD (1.45-fold) groups. Similarly, Mv-HFD-treated mice showed higher Glut4 expression than those in the CHD (1.36-fold), LFD (1.14-fold), and Mv-LFD (1.11-fold) groups; however, these differences were not statistically significant (Figure 6F).

Mice treated with an HFD exhibited higher gene expression of *Il6* compared to those in the CHD (1.89-fold), Mv-HFD (1.36-fold), LFD (1.85-fold), and Mv-LFD (1.57-fold) groups. However, these differences were not statistically significant (Figure 6G). HFD-treated mice showed significantly (*p *< 0.05) higher gene expression of *Tnfα* compared to those in the CHD (3.19-fold), Mv-HFD (1.98-fold), LFD (3.08-fold), and Mv-LFD (2.55-fold) groups (Figure 6H).

### 3.11. Mv-HFD and Mv-LFD Increased IWAT mRNA Expression of Ucp1, Cidea, and Cd137

Next, we investigated the effects of dietary fat content and vitamin supplementation on the gene expression of markers associated with IWAT browning. Mice treated with the Mv-HFD and Mv-LFD exhibited significantly (*p* < 0.05) higher gene expression of *Ucp1* compared to those in the CHD (3.36-fold and 3.44-fold) and HFD (2.73-fold and 2.79-fold) groups (Figure 6A). Mice treated with the Mv-HFD and Mv-LFD exhibited significantly (*p* < 0.05) higher gene expression of *Cidea* compared to those in the CHD (3.40-fold and 3.38-fold), HFD (2.37-fold and 2.35-fold), and LFD (2.40-fold and 2.38-fold) groups (Figure 6B). Similarly, the Mv-HFD- and Mv-LFD-treated mice showed significantly (*p* < 0.05) higher gene expression of *Cd137* compared to those in the CHD group (3.94-fold and 3.34-fold) (Figure 6C). Mice treated with the Mv-HFD and Mv-LFD exhibited higher gene expression of *Pgc1α* than those in the HFD group (2.11-fold and 2.10-fold). However, these differences were not statistically significant (Figure 6D).

We further investigated the effects of the Mv-HFD and Mv-LFD on the protein expression of the *Ucp1* browning marker in IWAT using IHC. Representative IHC images showed increased *Ucp1* staining in IWAT from mice treated with the Mv-HFD and Mv-LFD compared to controls treated with the CHD, HFD, and LFD (Figure 6E).

### 3.12. Mv-HFD and Mv-LFD Improved GM Composition at Phylum Level and α-Diversity

**Figure 7:**
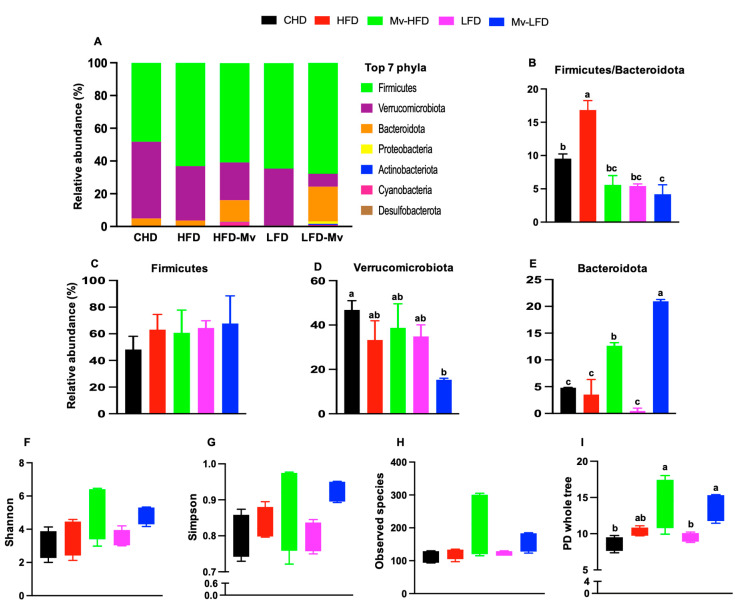
The Mv-HFD and Mv-LFD improved the gut microbiota composition at the phyla level and α-diversity compared to HFD-fed mice. (**A**) Composition of gut microbiota at the phylum level, (**B**) Firmicutes/Bacteroidetes, relative abundance of (**C**) Firmicutes, (**D**) Verrucomicrobiota, and (**E**) Bacteroidetes; boxplots based on the α-diversity indices (**F**) Shannon, (**G**) Simpson, (**H**) Observed species, and (**I**) PD whole tree. n = 2–3, data are presented as the mean ± SD. Bars or boxes without a common superscript differ, *p* < 0.05, via one-way ANOVA followed by Tukey’s HSD post hoc test.

As shown in Figure 7A, Firmicutes, Verrucomicrobiota, and Bacteroidota were the predominant phyla, collectively accounting for over 95% of the bacteria. The Firmicutes/Bacteroidota ratio was significantly (*p *< 0.01) higher in the HFD group compared to the CHD (1.76-fold), Mv-HFD (3.02-fold), LFD (3.11-fold), and Mv-LFD (4.01-fold) groups (Figure 7B).

The abundance of Firmicutes was higher in the HFD group than in the CHD (1.31-fold) and Mv-HFD (1.04-fold) groups, with percentages of 63%, 48%, and 60%, respectively. However, these differences were not statistically significant. Additionally, the LFD and Mv-LFD groups showed a higher abundance of Firmicutes (1.06-fold and 1.11-fold) than the Mv-HFD group, with 64% and 68% versus 60% (Figure 7C). The abundance of Verrucomicrobiota was lower in the Mv-HFD group compared to the CHD (3.05-fold, *p *< 0.05), HFD (2.17-fold), and LFD (2.27-fold) groups, with percentages of 15%, 47%, 33%, and 39%, respectively. However, the differences between the other groups were not statistically significant (Figure 7D). Additionally, the abundance of Bacteroidetes was significantly higher in the Mv-HFD (*p *< 0.05) and Mv-LFD (*p *< 0.001) groups compared to the CHD (2.63-fold and 4.35-fold), HFD (3.57-fold and 5.92-fold), and LFD (27.01-fold and 44.79-fold) groups. The Mv-LFD group also exhibited a higher abundance of Bacteroidetes compared to the Mv-HFD group (1.65-fold, *p *< 0.01) (Figure 7E).

The Shannon diversity index (Shannon), Simpson index (Simpson), observed species richness (observed species), and phylogenetic diversity whole tree (PD whole tree) were employed to evaluate the diversity of bacterial species. The Shannon and Simpson indices were used to assess diversity, while observed species richness characterized the overall richness. PD whole tree was utilized to evaluate evolution-based diversity. The Shannon index was higher in the Mv-HFD and Mv-LFD groups compared to the CHD (1.64-fold and 1.57-fold), HFD (1.44-fold and 1.37-fold), and LFD (1.49-fold and 1.43-fold) groups (Figure 7F). Similarly, the Simpson index was higher in the Mv-HFD and Mv-LFD groups compared to the CHD (1.10-fold and 1.15-fold), HFD (1.06-fold and 1.10-fold), and LFD (1.11-fold and 1.15-fold) groups (Figure 7G). The observed species richness was also greater in the Mv-HFD and Mv-LFD groups compared to the CHD (1.89-fold and 1.41-fold), HFD (1.72-fold and 1.28-fold), and LFD (1.73-fold and 1.28-fold) groups. However, these differences were not statistically significant (Figure 7H). In contrast, the PD whole tree was higher in the Mv-HFD and Mv-LFD groups compared to the CHD (1.66-fold and 1.59-fold, *p *< 0.05) and LFD (1.50-fold and 1.43-fold, *p *< 0.05) groups, while differences compared to the HFD group were not significant (Figure 7I).

### 3.13. Mv-HFD and Mv-LFD Altered GM Composition at Genus Level and Showed Slight Trend in Improvement in β-Diversity

**Figure 8:**
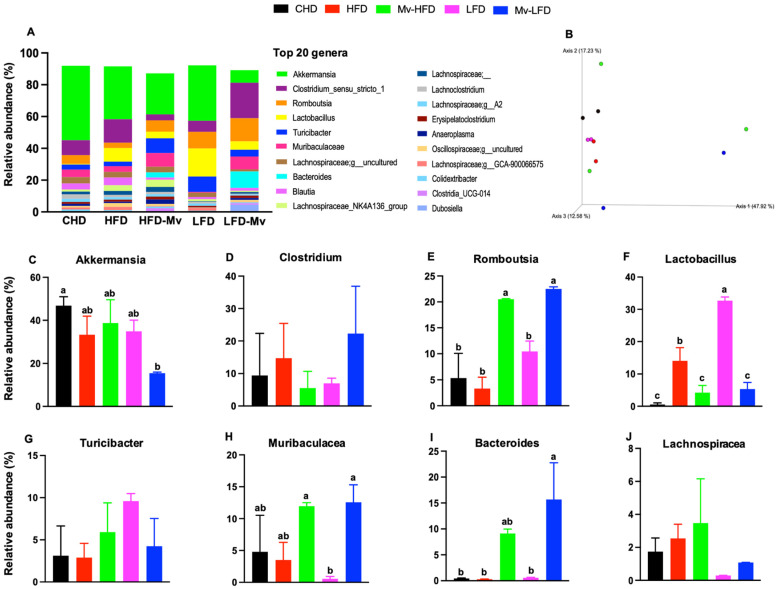
The Mv-HFD and Mv-LFD altered gut microbiota composition at the genus level and showed a slight trend in improving β-diversity compared to HFD-fed mice. (**A**) Composition of gut microbiota at the genus level, (**B**) a three-dimensional scatter plot, using principal coordinates analysis (PCoA) from Unweighted UniFrac analyses of β-diversity, relative abundance of (**C**) Akkermansia, (**D**) Clostridium, (**E**) Romboutsia, (**F**) Lactobacillus, (**G**) Turicibacter, (**H**) Muribaculaceae, (**I**) Bacteroides, and (**J**) Lachnospiraceae. n = 2–3, data are presented as the mean ± SD. Bars without a common superscript differ, *p* < 0.05, via one-way ANOVA followed by Tukey’s HSD post hoc test.

The most prevalent genera identified were Akkermansia, Clostridium, Romboutsia, Lactobacillus, Turicibacter, Muribaculaceae, Bacteroides, and Lachnospiraceae (Figure 8A). β-diversity was evaluated using unweighted and weighted UniFrac, Bray–Curtis, and Jaccard analyses. Distances were calculated among samples from various groups, revealing no significant differences (Figure 8B).

The Mv-HFD group exhibited a higher abundance of Akkermansia (1.16- and 1.11-fold), Romboutsia (6.18- and 1.96-fold, *p *< 0.05), Muribaculaceae (3.37- and 21.15-fold), Bacteroides (35.76- and 17.08-fold), and Lachnospiraceae (1.67- and 14.31-fold, *p *< 0.05) compared to the HFD and LFD groups (Figure 8C–J). The LFD group showed a significantly (*p *< 0.05) higher abundance of Lactobacillus compared to the CHD (59.28-fold), HFD (2.32-fold), Mv-HFD (9.10-fold), and Mv-LFD (6.11-fold) groups (Figure 8F).

The Mv-HFD group exhibited a higher abundance of several genera compared to the CHD group: Romboutsia (3.84-fold, *p *< 0.05), Lactobacillus (2.44-fold, *p *< 0.05), Turicibacter (1.29-fold), Muribaculaceae (2.46-fold), Bacteroides (21.45-fold), and Lachnospiraceae (21.45-fold). No significant differences were observed in bacterial abundance at the genus level between the Mv-HFD and Mv-LFD groups. However, the Mv-LFD group showed slightly lower levels of Akkermansia (2.51-fold) and Lachnospiraceae (3.93-fold) and higher levels of Bacteroides (1.61-fold) compared to the Mv-HFD group (Figure 8C–J).

## 4. Discussion

This study investigated the impact of multivitamin supplementation with thermogenic potential on obesity-related metabolic parameters, adipose tissue browning, and GM composition in mice. Mice were fed either an HFD or LFD, supplemented with vitamins A, D, B_1_, B_5_, and C, and compared to control groups without supplementation. The results indicated that multivitamins reduced body weight and fat mass while improving metabolic health by modulating glucose, lipid, and energy metabolisms in HFD-fed mice. These benefits were linked to increased adipose tissue browning and changes in GM composition. Notably, metabolic parameters in the Mv-HFD group were similar to those in the CHD, LFD, and Mv-LFD groups, suggesting that multivitamins can mitigate some negative effects of an HFD. However, no significant differences were found between the Mv-HFD and Mv-LFD groups, indicating consistent effects across dietary fat levels.

Dietary composition affects food intake patterns, with an HFD potentially decreasing consumption levels due to its higher calorie density [6,45,46]. In our comparative analysis, vitamin supplementation did not significantly change food intake among different diet groups, indicating consistent consumption levels between the supplemented and control diets.

Vitamin A and its derivatives are vital for many physiological functions, including development and immune regulation [47,48]. Recent studies revealed their significant effects on cardiometabolic health, particularly in adipogenesis and energy metabolism. While animal studies have shown positive results regarding obesity and metabolic health, human research has been inconsistent. This highlights the need for further investigation into the mechanisms by which vitamin A affects human physiology. Understanding these mechanisms is crucial, as vitamin A-targeted therapies for metabolic disorders are being explored, indicating a promising area for future clinical research [49].

Several studies using rodent models have investigated the link between dietary vitamin A or retinoic acid (RA) supplementation and obesity-related factors [30,50,51,52,53]. Vitamin A supplementation (129 mg/kg of diet, over 20 times higher than the current study’s dosage) in obese rats caused significant weight loss and reduced WAT compared to controls on a 2.6 mg/kg diet. This higher dose did not decrease food intake or cause vitamin A toxicity. While hypertriglyceridemia was seen in the supplemented rats, this correlation was not observed in the supplemented mice in the current study, likely due to differing dosages [50]. High doses of RA at 100 mg/kg body weight in NMRI mice and 15 mg slow-release RA pellets in obese mice (15 and 2.5 times higher than the current dosage, respectively) led to reduced body weight, fat mass, and serum triglycerides while improving glucose tolerance and insulin levels. These treatments also altered gene expression related to adipogenesis and thermogenesis, decreasing *Pparγ*, *Srebp1c*, and *Cebpα* and increasing *Ucp1* and *Pgc1α* in WAT [52,53], highlighting the importance of dosage and animal model selection in vitamin A supplementation research.

In contrast, dietary supplementation with vitamin A in a 320 mg/kg diet did not consistently impact body weight or adipose tissue mass in lean C57BL/6J mice [51]. Our study demonstrated consistent results using significantly lower doses compared to previous studies. We observed comprehensive improvements in metabolic parameters, including body weight, fat mass, reduced adipocyte size, liver lipid accumulation, enhanced energy expenditure, and improved glucose and lipid metabolisms in both the liver and WAT. Additionally, we noted a higher expression of browning markers such as *Ucp1*, *Cidea*, and *Cd137*. The significant effects observed with lower doses may be attributed to the synergistic effects of combining vitamin A with other vitamins in a multivitamin supplement, potentially enhancing its metabolic benefits while reducing each component’s required dose.

Vitamin A status significantly affects the GM composition in humans and mice [54,55]. Animal studies with vitamin A supplementation (20,000 IU/kg diet) have shown a decreased Firmicutes-to-Bacteroidetes ratio, which is consistent with our findings of reduced ratios, increased Bacteroidetes, and slight improvements in the α- and β-diversities in the supplemented groups. Notably, we observed increased levels of Muribaculaceae, Bacteroides, and Romboutsia [56,57]. These results highlight vitamin A’s crucial role in modulating GM composition, potentially enhancing metabolic health and weight management [58,59,60,61].

Vitamin D is crucial for calcium balance and bone health [62,63], with about 65% of vitamin D and 35% of 25(OH)D stored in adipose tissue. Adipocytes express vitamin D receptors (VDRs) and enzymes that influence metabolism, adipogenesis, lipid metabolism, and inflammation [64,65]. Research suggests that vitamin D supplementation may be less effective in obese individuals, with reduced benefits on lipid profiles and insulin resistance [66,67]. Those with low vitamin D levels or severe metabolic disorders may experience more significant advantages from supplementation [64,68].

Vitamin D supplementation in obese rodents has demonstrated significant metabolic benefits. Wistar rats receiving 800 to 2400 IU/kg diet of vitamin D showed reduced weight gain, less abdominal fat, and improved lipid profiles [46,69]. C57BL/6J mice given higher doses (10,000 to 15,000 IU/kg diet) experienced decreased inflammation in adipose tissue and reduced liver steatosis [70,71,72,73]. Additionally, a dose of 67 IU/kg body weight improved glucose transport in the adipose tissue of HFD-fed mice, indicating potential benefits for obesity-related glucose disorders [74]. However, another study suggests that glycemic control benefits may be limited to vitamin D-deficient obese mice [73]. These findings suggest vitamin D supplementation could enhance weight management and metabolic health in obese animal models.

The effect of vitamin D supplementation on adipose tissue browning in obese rodents has not been well studied. The VDR negatively regulates fat browning; its overexpression inhibits brown adipocyte differentiation and *Pparγ* activation by downregulating *Pgc1α* and *Prdm16*, which decreases the *Ucp1*, *Ucp2*, and *Ucp3* levels in BAT. Knocking down the VDR reverses these effects. Additionally, fat-specific VDR knockout in mice increases *Ucp1* expression in visceral adipose tissue, promoting energy expenditure and the browning of WAT [64]. Conversely, C57BL/6J mice fed an HFD supplemented with vitamin D (15,000 IU/kg diet) experienced lower weight gain and reduced adipose tissue mass. This was linked to increased energy expenditure and improved lipid utilization, as vitamin D enhanced the expression of genes related to energy metabolism, fatty acid oxidation, and mitochondrial biogenesis, including *Pgc1α* and *Cpt1* in BAT and muscle [33]. Our results confirmed the benefits of vitamin D supplementation on body weight, glucose, and lipid metabolism. Additionally, we observed a significant reduction in adipocyte size and increased the expression of browning markers in WAT. Notably, our study demonstrates that vitamin D supplementation can induce the browning of WAT, potentially enhancing energy expenditure in HFD-obese mice. Further metabolic improvements may be attributed to the synergistic effects of combining vitamin D with other vitamins in a multivitamin supplement, potentially amplifying its metabolic benefits.

It has been shown that an HFD significantly reduces the richness and diversity of microbial communities in obese mice. Consistent with our findings, vitamin D supplementation (5000 to 11,000 IU/kg diet) has been found to counteract these effects, improving microbiota diversity and altering bacterial composition. Specifically, it decreases Firmicutes abundance while enhancing Akkermansia and Bacteroidetes levels, helping to restore an HFD-altered GM [73]. In human studies, vitamin D supplementation has shown similar benefits [75,76].

Vitamin C is linked to a reduced risk of various health issues and obesity in both humans and animals [34]. Its potential mechanisms include affecting adipocyte lipolysis, improving glucose metabolism, and reducing inflammation, all of which are likely connected to its antioxidant properties [34].

In obese ovariectomized C57BL/6J mice, a high dose of ascorbic acid (5000 mg/kg diet) led to smaller adipocytes, reduced inflammation, and improved glucose tolerance compared to untreated mice [77]. Similarly, ob/ob mice given vitamin C (25 g/L in drinking water) had lower plasma glucose and insulin levels than untreated controls [78]. In another study, male wild-type C57BL/6J mice on an HFD supplemented with vitamin C (600 mg/kg body weight) lost weight and fat mass and had smaller adipocytes. These effects were linked to the reduced expression of lipogenic enzymes (*Fas*, *Srebp1c*, and *Pparγ*) in adipose tissue, along with lower fasting glucose levels and improved glucose tolerance [79]. In a study similar to ours, C57BL/6J mice were fed an LFD, HFD, or HFD with ascorbic acid (1000 mg/kg) for 15 weeks. Vitamin C supplementation resulted in less body weight gain, reduced fat mass, smaller adipocyte sizes, and lower inflammation and cholesterol in the liver compared to the HFD group [80]. Our multivitamin supplementation, given at the same dosage for a shorter duration, resulted in 1.39 times less body weight gain and 7 times reduction in adipocyte size compared to the HFD group. This suggests the benefits are due to the combined effects of multivitamins rather than just vitamin C. Studies consistently show that vitamin C supplementation can help reduce the impact of an HFD on body weight, adipocyte size, and metabolic health in mouse models. Additionally, it has been suggested that vitamin C supplementation (25 mg/kg body weight) may reduce weight in rats by activating interscapular BAT through enhanced glucose and fatty acid uptake and increased thermogenesis. However, vitamin C’s effects on WAT’s browning have not been extensively studied. This study is the first to assess vitamin C and other browning-promoting vitamins in mice, revealing the increased expression of *Ucp1* and other browning markers in WAT, along with a reduced RER, suggesting a shift towards greater lipid oxidation over carbohydrate metabolism [36].

Consistent with our findings, a previous study has shown that vitamin C supplementation at 30 mg/kg body weight/day can reverse the altered abundances of key GM phyla, including Firmicutes and Bacteroidetes, in HFD-induced obese C57BL/6J mice. This supplementation also affected specific genera such as Muribaculaceae, Lachnospiraceae, and Bacteroides. Additionally, the combination of vitamins C and D enhanced GM diversity and richness, as indicated by the α-diversity analysis, underscoring their vital role in regulating GM composition [81]. Furthermore, it has been demonstrated that a dosage of 500 mg/day of vitamin C increased the α-diversity in humans compared to baseline and placebo conditions [82].

Vitamin B_1_ is a key cofactor in glycolysis, ATP production, and the pentose phosphate pathway, supporting energy metabolism and synthesizing cellular components and neurotransmitters [83]. Vitamin B_1_ deficiency disrupts glucose metabolism, energy production, and macronutrient processing, leading to lactic acidosis and decreased ATP synthesis. It is closely linked to chronic diseases like obesity, where thiamin deficiency is more common and contributes to insulin resistance and impaired glucose tolerance [83,84].

Vitamin B_1_ supplementation has been shown to prevent obesity and metabolic disorders in both humans and rodents [85,86,87]. In obese rats, a 0.2% *w*/*v* dose (4 times higher than the current study’s dosage) enhanced lipid oxidation and reduced adipocyte hypertrophy and liver steatosis. Thiamine treatment also preserved cardiac and renal functions, highlighting its potential benefits for obesity and metabolic health [87]. A study on non-insulin-dependent diabetic KK mice examined a mixture of thiamin, arginine, caffeine, and citric acid for its anti-obesity effects. The mixture significantly reduced adipose tissue weight, liver lipid content, and plasma insulin levels. When combined with a low-calorie diet, it showed the potential to improve lipid metabolism and reduce body fat [86]. Similar results were observed in healthy individuals with high body fat [85]. In line with these findings, our study indicated that multivitamin supplementation containing vitamin B_1_ also decreased body weight, fat gain, adipocyte size, liver lipid accumulation, and improved glucose tolerance in HFD-fed obese mice.

An in vitro study found that *Ucp1*-enriched human neck adipocytes rely on thiamine via the thiamine transporter (ThTr2) during cAMP-induced thermogenic activation. Blocking ThTr2 disrupts thiamine uptake, uncoupling, and proton leak respiration, while thiamine or thiamine pyrophosphate (TPP) restores these functions by supporting pyruvate dehydrogenase. ThTr2 inhibition also impairs thermogenic gene activation (e.g., *Ucp1*, *Pgc1α*), whereas thiamine enhances their expression, highlighting its key role in thermogenesis and potential for obesity prevention or treatment [37,38]. No previous study has examined vitamin B_1_ supplementation’s effects on thermogenesis and metabolic enhancement in obesity. This study found that multivitamin supplementation with thiamine improved adipose tissue browning, enhanced energy metabolism, reduced the RER (indicating greater lipid utilization), and enhanced cold tolerance, likely via increased *Ucp1* activity. These findings align with research showing thiamine promotes uncoupling and thermogenic gene expression in *Ucp1*-enriched adipocytes, highlighting its potential as a therapeutic for obesity prevention and treatment.

Supplementing with vitamin B_1_ has been shown to modify the GM in both humans and mice. Similar to our findings, increased B_1_ intake raised the levels of Bacteroidetes and Verrucomicrobiota [88]. In a study with male C57BL/6 mice on an obesogenic diet, a 1 mg/kg dose reduced weight gain and increased the Lachnospiraceae family from the Firmicutes phylum [89]. These results suggest that thiamine supplementation may help regulate body weight and alter GM composition.

Vitamin B_5_ is essential for cellular energy metabolism and preventing metabolic disorders [90]. Pantothenic acid (PA) converts into coenzyme A (CoA), which is necessary for synthesizing fatty acids, cholesterol, and acetylcholine. CoA also helps produce adenosine triphosphate in mitochondria using fatty acids, carbohydrates, and amino acids. While PA is used as a dietary supplement for nutrient deficiencies, its role in obesity and metabolic conditions is still debated [91]. It has been suggested that a high intake of vitamin B_5_ and other fortified B vitamins may be linked to increased obesity and metabolic disorders in children and adolescents [91,92]. Conversely, a study found that PA ingestion negatively correlated with the visceral fat area [93]. Another study showed that administering pantethine, a CoA production intermediate, lowered cardiovascular risk markers like LDL and total cholesterol without significantly changing the BMI [94].

Limited in vitro and in vivo studies have investigated the effects of vitamin B_5_ on adipose tissue browning. A study on chemical compound-induced brown adipocytes found that low concentrations of PA (0.25 and 1 μg/mL) significantly induced thermogenic genes *Ucp1* and *Cidea* expression, while higher concentrations (4 and 16 μg/mL) suppressed them. The same study also showed that vitamin B_5_ uniquely regulates thermogenic gene expression by affecting glucose and lipid metabolisms [91]. Treatment with PA at 1, 5, and 25 µM significantly increased *Ucp1* expression in human primary brown adipocytes. Additionally, 10 mg/kg body weight/day PA supplementation for 11 weeks in HFD-induced obese C57BL/6J mice reduced body weight gain, decreased adipose tissue weight, and increased *Ucp1* expression [21]. Our study confirms that vitamin B_5_ supplementation improves obesity-related metabolic parameters, such as reducing body weight gain and fat mass while enhancing lipid and glucose metabolisms. These benefits, likely due to increased energy expenditure and adipose tissue browning, were most effective when vitamin B_5_ was taken with other browning vitamins.

Current research on vitamin B_5_ supplementation’s impact on the GM is limited [95]. It has been previously shown that higher vitamin B_5_ intake increased Prevotella and Actinobacteria while reducing Bacteroides in lactating women [96] and had non-linear effects on microbiota diversity [97]. A diet with 26.0 mg/kg of vitamin B5 in fish increased microbiota diversity and abundance [98]. An in vitro study of L. helveticus showed that a vitamin B_5_-deficient medium significantly inhibited fatty acid and protein synthesis, likely due to the downregulation of genes related to these processes [99]. Overall, vitamin B_5_ deficiency may disrupt GM growth and function [99]. Since no previous studies have specifically examined the effects of B_5_ supplementation on GM composition in obese mice, we cannot compare the current findings directly. However, our results confirmed improvements in GM composition and diversity and increased beneficial bacteria, aligning with previous research suggestions.

The dosage used in this study was based on mouse models’ highest reported safe levels, and no toxicity was observed at the supplemented doses. Our findings suggest that multivitamin formulations aimed at promoting adipose tissue browning are both safe and promising, potentially offering a novel approach to combat obesity and improve metabolic health. Inducing adipose tissue browning in humans through specific micronutrient intake presents a promising long-term strategy for managing obesity and related metabolic disorders. However, it is important to note that nutritional intervention strategies are still in the early stages of development and require further research.

## 5. Conclusions

In conclusion, this study demonstrates the potential of multivitamin supplementation in modulating metabolic health and GM composition. The upregulation of browning markers in adipose tissue of mice receiving multivitamin-supplemented diets suggests a promising approach to combatting obesity through dietary intervention. The observed shifts in the GM, particularly the increased abundance of beneficial bacteria, underscore the broad impact of nutritional supplementation on health. These findings highlight the complex interplay between diet, metabolism, and gut health, opening new avenues for targeted nutritional strategies to address obesity and related disorders. While encouraging, further research is needed to elucidate the underlying mechanisms and translate these findings to human interventions. Future research should optimize nutritional interventions and explore their long-term health effects to develop innovative strategies against obesity and its metabolic complications.

## Data Availability

The data supporting this study’s findings are available from the corresponding author upon reasonable request. The data will be used for future analysis and the development of other ongoing projects.

## Supplementary Materials

The following supporting information can be downloaded at https://www.mdpi.com/article/10.3390/nu17061045/s1: Table S1: Compositions of the custom diets; Table S2: Primer sequences.

## Abbreviations

AUC	Area under the curve
BAT	Brown adipose tissue
VCO_2_	Carbon dioxide production
Cidea	Cell death-inducing DNA fragmentation factor-like effector A
Cd137	Cluster of differentiation 137
Cebpα	CCAAT/enhancer-binding protein alpha
CHD	Control chow diet
CoA	Coenzyme A
EE	Energy expenditure
Fabp4	Fatty acid-binding protein 4
Fas	Fatty acid synthase
GTT	Glucose tolerance test
Glut2	Glucose transporter 2
Glut4	Glucose transporter 4
GWAT	Gonadal white adipose tissue
GM	Gut microbiome
H&E	Hematoxylin and Eosin
HFD	High-fat diet
Il6	Interleukin 6
IWAT	Inguinal white adipose tissue
HOMA-IR	Homeostatic model assessment for insulin resistance
LFD	Low-fat diet
Mv-HFD	Multivitamin-supplemented HFD
Mv-LFD	Multivitamin-supplemented LFD
VO_2_	Oxygen consumption
PA	Pantothenic acid
Pgc1α	Peroxisome proliferator-activated receptor gamma coactivator 1-alpha
Pparγ	Peroxisome proliferator-activated receptor gamma
Prdm16	PR domain containing 16
PCoA	Principal coordinates analysis
T_Core_	Rectal temperature
RER	Respiratory exchange ratio
RA	Retinoic acid
Srebp1c	Sterol regulatory element-binding protein 1c
T_Skin_	Surface temperature
ThTr2	Thiamine transporter
Tnfα	Tumor necrosis factor alpha
Ucp1	Uncoupling protein 1
V4	Variable region 4
WAT	White adipose tissue
