# Supplementary material for: Effects of Multivitamin Supplementation on Metabolic Parameters in High- and Low-Fat Diet-Fed C57BL/6J Mice: Potential Links to Adipose Tissue Browning and Gut Microbiome
Source: Nutrients. 2025 Mar 17;17(6):1045. doi: 10.3390/nu17061045 (PMC11944532; doi:10.3390/nu17061045)
Supplement: Supplementary file 1 [file nutrients-17-01045-s001.zip › nutrients-3507726-supplementary.pdf]

D12450K, D12451, D23112201 and D23112202

Formulated by
Shailee Jani
Research Diets Inc

Rodent Diets With 10 or 45 kcal% Fat and Same With added Vitamins A, D3, B1, B5, and C

Table S1: Composition of the custom diets

**Product #**	**D12450K**		**D23112201**		**D12451**		**D23112202**	
	10 kcal% Fat Control		*10 kcal% Fat with vitamins*		*45 kcal% Fat Control*		*45 kcal% Fat with vitamins*	
	**gm%**	***kcal%***	**gm%**	***kcal%***	**gm%**	***kcal%***	**gm%**	***kcal%***
**Protein**	**19.2**	***20***	**19.2**	***20***	**23.7**	***20***	**23.5**	***20***
**Carbohydrate**	**67.3**	***70***	**67.0**	***70***	**41.4**	***35***	**41.2**	***35***
**Fat**	**4.3**	***10***	**4.2**	***10***	**23.6**	***45***	**23.5**	***45***
**Total**		***100***		***100***		***100***		***100***
**kcal/gm**	**3.85**		**3.83**		**4.73**		**4.70**	
**Ingredient**	**gm**	***kcal***	**gm**	***kcal***	**gm**	***kcal***	**gm**	***kcal***
Casein	200	*800*	200	*800*	200	*800*	200	*800*
L-Cystine	3	*12*	3	*12*	3	*12*	3	*12*
Corn Starch	550	*2200*	550	*2200*	72.8	*291.2*	72.8	*291.2*
Maltodextrin 10	150	*600*	150	*600*	100	*400*	100	*400*
Sucrose	0	*0*	0	*0*	172.8	*691*	172.8	*691*
Cellulose, BW200	50	*0*	50	*0*	50	*0*	50	*0*
Soybean Oil	25	*225*	25	*225*	25	*225*	25	*225*
Lard	20	*180*	20	*180*	177.5	*1597.5*	177.5	*1597.5*
Mineral Mix S10026	10	*0*	10	*0*	10	*0*	10	*0*
DiCalcium Phosphate	13	*0*	13	*0*	13	*0*	13	*0*
Calcium Carbonate	5.5	*0*	5.5	*0*	5.5	*0*	5.5	*0*
Potassium Citrate, 1 H2O	16.5	*0*	16.5	*0*	16.5	*0*	16.5	*0*
Vitamin Mix V10001	10	*40*	10	*40*	10	*40*	10	*40*
Choline Bitartrate	2	*0*	2	*0*	2	*0*	2	*0*
**Vitamin A Acetate (500,000 IU/gm)**	0	*0*	0.04	0	*0*	*0*	0.04	*0*
**Vitamin D3 (100,000 IU/gm)**	0	0	0.25	0	0	0	0.25	0
**Vitamin B1 (Thiamine HCl 78.6%, active)**	0	0	0.636	0	0	0	0.636	0
**Vitamin B5 (Calcium Pantothenate)**	0	0	0.5	0	0	0	0.5	0
**Vitamin C (Ascorbic Acid Phosphate, L)**	0	0	3.03	0	0	0	3.03	0
FD&C Blue Dye #1	0.025	*0*	0.05	*0*	0	*0*	0.025	*0*
FD&C Yellow Dye #5	0	*0*	0	*0*	0	*0*	0.025	*0*
FD&C Red Dye #40	0.025	*0*	0	*0*	0.05	*0*	0	*0*
**Total**	**1055.05**	***4057***	**1059.506**	***4057***	**858.15**	***4057***	**862.606**	***4057***
	**D12450K**		**D23112201**		**D12451**		**D23112202**	
**Added Levels of Vitamins (on top of existing levels)**								
**Vitamin A (IU)**	**0**		**20000**		**0**		**20000**	
**Vitamin D3 (IU)**	**0**		**25000**		**0**		**25000**	
**Vitamin B1 (mg)**	**0**		**500**		**0**		**500**	
**Vitamin B5 (mg)**	**0**		**500**		**0**		**500**	
**Vitamin C (mg)**	**0**		**1000**		**0**		**1000**	
**Final IU or ug/Kcal**								
**Vitamin A (IU)**	1.0		5.9		1.0		5.9	
**Vitamin D3 (IU)**	0.2		6.4		0.2		6.4	
**Vitamin B1 (ug)**	1.2		124.4		1.2		124.4	
**Vitamin B5 (ug)**	3.9		127.2		3.9		127.2	
**Vitamin C (ug)**	0.0		246.5		0.0		246.5	
**Estimated Levels of Vitamins**								
**Vitamin A (IU/kg)**	**3791**		**22652**		**4661**		**27823**	
**Vitamin D3 (IU/kg)**	**948**		**24540**		**1165**		**30141**	
**Vitamin B1 (mg/kg)**	**4**		**476**		**6**		**585**	
**Vitamin B5 (mg/kg)**	**15**		**487**		**19**		**598**	
**Vitamin C (mg/kg)**	**0**		**944**		**0**		**1159**	

Vitamin and mineral levels are based on the amounts added to the diets and do not take into account any residual levels in ingredients. If you need to know the exact levels of vitamins and minerals, we suggest that they be assayed prior to beginning your study.

Table S2. Primer sequences

36b4	**Forward**	GCTTCGTGTTCACCAAGGAGGA
	**Reverse**	GTCCTAGACCAGTGTTCTGAGC
β-actin	**Forward**	GTGACGTTGACATCCGTAAAGA
	**Reverse**	GCCGGACTCATCGTACTCC
Cebpα	**Forward**	GCAAAGCCAAGAAGTCGGTGGA
	**Reverse**	CCTTCTGTTGCGTCTCCACGTT
Cd137	**Forward**	CGTGCAGAACTCCTGTGATAAC
	**Reverse**	GTCCACCTATGCTGGAGAAGG
Cidea	**Forward**	GGTGGACACAGAGGAGTTCTTTC
	**Reverse**	CGAAGGTGACTCTGGCTATCC
Fas	**Forward**	GACCTCAGGCTGCAGTGAAT
	**Reverse**	GTCCCCACTTGATGTGAGGGG
Fabp4	**Forward**	TGA AATCACCGCAGACGACAGG
	**Reverse**	GCTTGTCACCATCTCGTTTTCTC
G6p	**Forward**	AGATAGCAAGAGTAGAAGTGAC
	**Reverse**	TTCCAAGTGGATTCTGTTTGG
Glut2	**Forward**	TGAGTTCCTTCCAGTTCGGC
	**Reverse**	CTGAGGCCAGCAATTCTGACT
Glut4	**Forward**	GCTCTGACGTAAGGATGGGG
	**Reverse**	TGGCCAGTTGGTTGAGTGTT
Il6	**Forward**	GTTCTCTGGAAATCGTGGA
	**Reverse**	GCCACTCCTTCTGTGACTCC
Pgc1α	**Forward**	GAATCAAGCCACTACAGACACCG
	**Reverse**	CATCCCTCTTGAGCCTTTCGTG
Pparγ	**Forward**	GTACTGTCGGTTTCAGAAGTGCC
	**Reverse**	ATCTCCGCCAACAGCTTCTCCT
Srebp1c	**Forward**	CTGGTGAGTGGAGGGACCAT
	**Reverse**	GAGCCATCTCTGCTCTCTGC
Tnfa	**Forward**	TGTAGCCCACGTCGTAGCAAA
	**Reverse**	GCTGGCACCACTAGTTGGTTGT
Ucp1	**Forward**	GCTTTGCCTCACTCAGGATTGG
	**Reverse**	CCAATGAACACTGCCACACCTC
